# Standardisation and international adoption of defined approaches for skin sensitisation

**DOI:** 10.3389/ftox.2022.943152

**Published:** 2022-08-11

**Authors:** Silvia Casati, David Asturiol, Patience Browne, Nicole Kleinstreuer, Michèle Régimbald-Krnel, Pierre Therriault

**Affiliations:** ^1^ European Commission, Joint Research Centre (JRC), Ispra, Italy; ^2^ OECD Environment Directorate, Environment Health and Safety Division, Paris, France; ^3^ NIH/NIEHS/DNTP/The NTP Interagency Center for the Evaluation of Alternative Toxicological Methods, Research Triangle Park, Durham, NC, United States; ^4^ Environmental Health Science and Research Bureau, Healthy Environments and Consumer Safety Branch, Health Canada, Ottawa, ON, Canada; ^5^ Health Effects Division, Pest Management Regulatory Agency, Health Canada, Ottawa, ON, Canada

**Keywords:** defined approaches, international harmonisation, mechanistic relevance, adverse outcome pathway, non-animal alternative methods

## Abstract

In the absence of stand-alone one-to-one replacements for existing animal tests, efforts were made to integrate data from *in silico*, *in chemico* and *in vitro* methods to ensure sufficient mechanistic coverage of the skin sensitisation Adverse Outcome Pathway (AOP) and generate predictions suitable for hazard identification and potency sub-categorisation. A number of defined approaches (DAs), using fixed data interpretation procedures (DIP) to integrate data from multiple non-animal information sources, were proposed and documented using a standard reporting template developed by the Organisation for Economic Co-operation and Development (OECD). Subsequent international activities focused on the extensive characterisation of three of these DAs with respect to the reference *in vivo* data, applicability domains, limitations, predictive performances and characterisations of the level of confidence associated with the predictions. The ultimate product of this project was an OECD Guideline that provides information equivalent to that provided by the animal studies and that can be used to satisfy countries’ regulatory data requirements for skin sensitisation. This Defined Approach Guideline was the first of its kind for the OECD, and provides an important precedent for regulatory adoption of human biology-relevant new approach methodologies with performances equivalent to, or better than, traditional animal tests. This mini review summarizes the principal features of the defined approaches described in OECD guideline 497.

## Introduction

*In chemico* and *in vitro* test methods addressing the first three key events (KE) of the skin sensitisation adverse outcome pathway (AOP) (OECD 2012) have been adopted by the Organisation for Economic Co-operation and Development (OECD) as KE-based test guidelines (OECD 2022a; 2022b; 2022c). These methods, when used in isolation, are not able to address all regulatory requirements on the skin sensitisation potential and potency of chemicals comparable to that provided by traditional animal tests. Defined approaches (DAs), integrating data from various non-animal information sources in a specific combination and using fixed data interpretation procedures to derive a prediction of *in vivo* response, overcome some of the limitations of the individual tests and avoid expert judgment in the derivation of the prediction. As a first step towards international harmonisation, twelve defined approaches were described in OECD Guidance Document 256, (OECD 2016), and a preliminary assessment was published in Kleinstreuer et al. (Kleinstreuer et al., 2018). Later, based on a proposal supported by the International Cooperation on Alternative Test Methods (ICATM) (Casati et al., 2018), the project to develop a guideline on DAs was included in the OECD work program. After 3 years of intensive evaluation by the lead organisations, i.e. the US Environmental Protection Agency, the European Commission and Health Canada, supported by the OECD Secretariat and a “Defined Approaches for Skin Sensitisation Expert Group” (DASS EG) of scientific experts from regulatory agencies, validation bodies, non-governmental organisations and industry; Guideline 497 (OECD 2021a) was finally adopted.

Three DAs are included in the guideline: the “2 out of 3" defined approach (Bauch et al., 2012; Urbisch et al., 2015), the integrated testing strategy version 1 (ITSv1) (Takenouchi et al., 2015) and the integrated testing strategy version 2 (ITSv2). All of them are based on the use of OECD test guideline methods, namely the direct peptide reactivity assay (DPRA) (OECD 2022a), the KeratinoSens™ (OECD 2022b), and the human cell line activation test (h-CLAT) (OECD 2022c), for which reliability i.e. transferability and within- and between-laboratory reproducibility, have been characterised during the validation phase (Casati et al., 2013, 2014; Joint Research Centre, 2015). The ITSv1 and ITSv2 also make use of an *in silico* information source provided either by Derek Nexus (ITSv1), or OECD QSAR Toolbox (ITSv2).

The three DAs provide information for hazard identification according to the UN Globally Harmonized System of Classification and Labelling of Chemicals (United Nations, 2019). In addition, the ITSv1 and ITSv2 DAs can be used for allocating chemicals into potency sub-categories (UN GHS; Category 1A = strong sensitisers; Category 1B = other sensitisers), a long-awaited feature of alternative methods intended for regulatory use.

**FIGURE 1:**
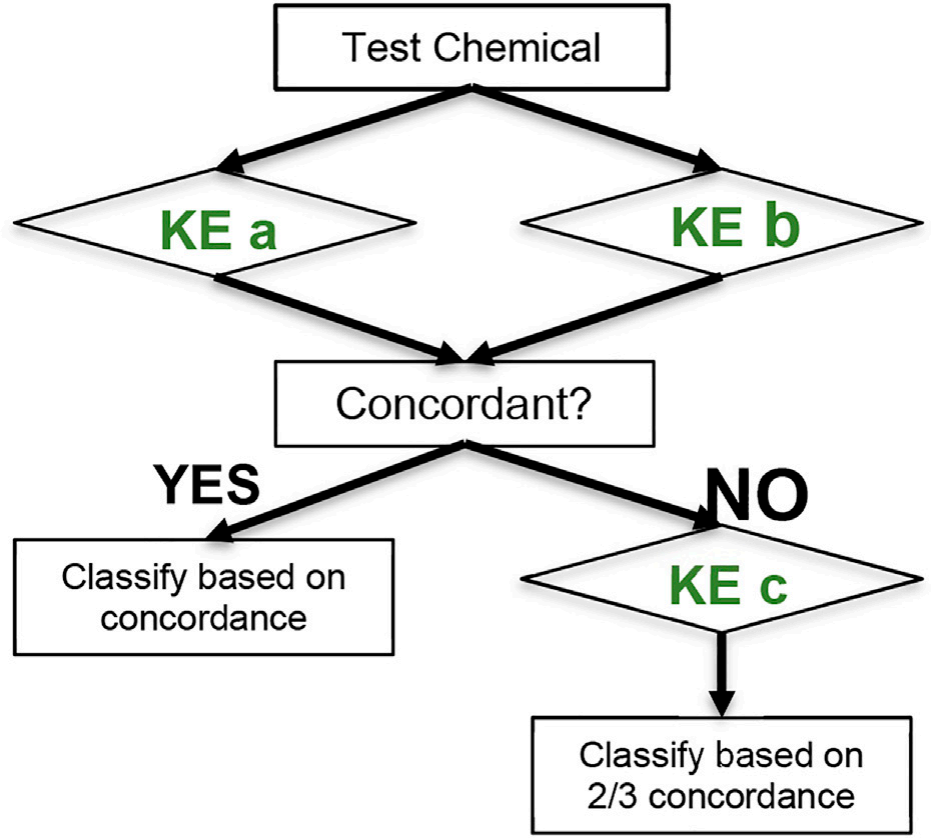
Decision tree applied in the 2 out of 3 (2o3) defined approach. In the 2o3 defined approach concordant predictions for two Key Event (KE) allow to classify a chemical as sensitiser or non-sensitiser. In case of discordant results, information on a third KE needs to be generated to conclude.

The 2 out of 3 (2o3) DA involves the generation of data to cover at least two of the three key events of the AOP by running, in an undefined order, the DPRA, KeratinoSens™ and h-CLAT (OECD 2021a). The DPRA generates information on the first KE of the AOP, covalent binding to proteins, by quantifying the reactivity of test chemicals towards model synthetic peptides containing either lysine or cysteine (OECD 2022a). The KeratinoSens™ addresses the activation of keratinocytes, the second KE, by measuring the Nrf2-mediated activation of antioxidant response element (ARE)-dependent genes via a luciferase readout (OECD 2022b). Information on KE3 is provided by the h-CLAT through quantification of the change in the expression of CD84 and CD54 cell surface markers associated with the process of activation of monocytes and dendritic cells (OECD 2022c). The 2o3 uses as source information for its prediction the predictions from the individual assays it is composed of (sensitiser/non-sensitiser). If two of the three assays provide a consistent prediction, the chemical is classified accordingly by the 2o3. If the individual assays are run sequentially and the first two assays have a discordant outcome, then a third assay needs to be conducted to draw a conclusion (Figure 1).

**FIGURE 2:**
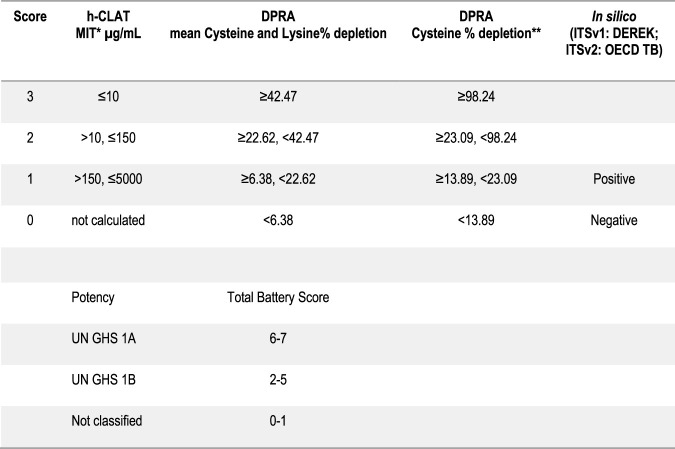
Data interpretation procedure used in the ITSv1 and ITSv2. *Minimum Induction Threshold (MIT). From the experimental concentration-response curves, the median concentration(s) inducing 1.5- and/or 2-fold induction of CD86 and/or CD54 are calculated and the lower of the two values is defined as the MIT. *Cysteine-only depletion thresholds are used in the case of co-elution with the lysine peptide. UN GHS 1A correspond to strong sensitisers and UN GHS 1B correspond to other (moderate to weak) sensitisers. Not classified are considered non-sensitisers.

The ITSv1 and ITSv2 use a score-based system to convert quantitative readouts generated with DPRA, h-CLAT and *in sili*co predictions from either Derek Nexus (Chilton et al., 2018) or the OECD QSAR Toolbox (TB) (Yordanova et al., 2019) into a final hazard or potency (UN GHS sub-categories 1A and 1B) prediction (Figure 2).

A total battery score of 2 or greater identifies skin sensitisers (UN GHS Cat. 1), a score of 6-7 corresponds to strong (UN GHS Cat. 1A) sensitiser, a score of 2-5 to moderate/weak (UN GHS Cat. 1B) sensitiser and a score of 1 or 0, as not classified (i.e. a non-sensitiser) (OECD 2021a).

The performance of the DAs described in this guideline for discriminating between sensitisers and non-sensitisers was evaluated using 168 test chemicals (135 GHS Skin Sens. Category 1, and 33 no classification) with DPRA, KeratinoSens™, h-CLAT, Derek, and OECD QSAR TB predictions, and expert group consensus classifications based on curated local lymph node assay (LLNA) reference data. Of these chemicals, 56 also had human reference classifications that were similarly examined and agreed upon by the DASS EG, as described below. For evaluating the performance of the ITS DAs for predicting UN GHS potency categorization (sub-category 1A, 1B, or “not classified” (NC)), 156 test chemicals (38 1A, 85 1B, and 33 NC based on LLNA data) were used, and 47 of these had human reference potency categories.

## *In vivo* reference data curation

The set of *in vivo* reference data used to assess the DAs performance underwent extensive curation (OECD 2021b).

For the available LLNA studies collected through different sources, a set of criteria for inclusion/exclusion were agreed upon. These criteria were based on the essential test method components of the LLNA Performance Standards for the validation of modifications to the traditional LLNA as described in OECD TG 429 (OECD, 2010). According to these criteria, the test chemical must be applied topically to both ears of the mice, lymphocyte proliferation must be measured during the induction phase of skin sensitisation and in the draining lymph nodes at the site of test chemical application, a vehicle control must be included in each study, and either individual or pooled animal data may be collected. Additional inclusion criteria considered were: the availability of concentrations tested and corresponding stimulation index (SI) values, *in vivo* administration of 3H-methyl thymidine or other radiolabelled markers, and that sodium dodecyl sulphate (SDS) was not applied as pre-treatment. Mixtures and botanicals with undefined structural composition were excluded from the curated LLNA reference data.

Furthermore, a minority of LLNA results had an Estimated Concentration at three-fold SI (EC3) outside the measured dose range (i.e. the EC3 values were extrapolated). To determine the reliability of the extrapolated EC3 and its suitability for UN GHS sub-categorisation (UN GHS sub-category 1A vs. sub-category 1B), three criteria, partially based on Ryan et al. (2007), were developed and consistently applied to the relevant LLNA studies. The following had to be met: 1) the extrapolated EC3 was less than ten-fold smaller than the closest tested concentration, 2) the lowest measured SI value was less than five and 3) the curve slope ratio was less than two. Details on the interpretation of the extrapolated EC3 values are reported in Annex 3 to the Supporting Document to the OECD Guideline 497 (OECD 2021b).

Specific considerations were also given to negative LLNA results for chemicals not tested up to a concentration of 100% v/v or w/v as recommended by OECD GL 429, and without a documented scientific rationale for the maximum test concentration selected. Study results were accepted as a reliable negative only if for all test concentrations SI values were < 3, and if the test chemical was tested at a concentration of at least 50%. Negative study results were also accepted if a valid scientific reason (in accordance with TG 429) was provided explaining why the highest tested concentration was lower than 50%. When multiple LLNA test results were available for the same chemical, the Median Like Location Parameter (MLLP) approach to derive an overall reference classification was applied (Hoffmann et al., 2018).

By applying these criteria, an unambiguous classification based on LLNA reference data was obtained for 168 of the chemicals, of which 135 were classified as sensitisers (UN GHS Skin Sens. 1) and 33 were not classified (NC). For 123 chemicals with an unambiguous classification as skin sensitisers, a UN GHS sub-categorisation could also be obtained. Of these, 38 were classified as Un GHS 1A and 85 as UN GHS 1B.

The same rigorous approach was applied in the review of the available Human Predictive Patch Test (HPPT) data. To that end, a database of HPPT studies was built starting from the information collated previously at the United States National Toxicology Program Interagency Center for the Evaluation of Alternative Toxicological Methods (NICEATM) and complemented with test results from the scientific literature, most of which were monographs on fragrance ingredients published by the Research Institute for Fragrance Materials (RIFM). The HPPT database, available via NICEATM’s Integrated Chemical Environment (https://ice.ntp.niehs.nih.gov/), contains information on chemical identity, test design, and test results and provides bibliographic information on the original test reports.

Review of the data included an analysis of the sources of variability and uncertainty, and assignment of relative reliability scores to the respective studies. In addition, decision logic for the classification of substances according to the UN GHS was developed and applied to the studies. Human data suitable for hazard classification was available for 66 chemicals of which 55 were classified as sensitisers and 11 were not classified (considered to be non-sensitisers). For the sensitisers, potency sub-categorisation could be obtained for 52 chemicals, 21 were classified as UN GHS 1A and 31 as UN GHS 1B.

## Establishing a level of confidence in the defined approaches predictions

The *in chemico* and *in vitro* methods used in the DAs employ a prediction model based on cut-offs to discriminate between sensitising and non-sensitising chemicals. In the DPRA, a test chemical is considered a sensitiser if it induces a mean peptide depletion of cysteine- and lysine-containing peptides above 6.38% (or in the case of co-elution, a cysteine-only % depletion above 13.89%) (OECD 2022a). In the KeratinoSens™ the criterion for a positive result is a luciferase fold induction >1.5 with cell viability > 70% when compared to the vehicle control (OECD 2022b). The h-CLAT considers a test chemical to be positive if the CD86 induction exceeds 1.5 fold and/or CD54 exceeds 2-fold at viabilities > 50% when compared to the vehicle control (OECD 2022c). For all assays, any result close to the cut-off tends to be less certain due to the higher incidence of false negative and false positive predictions. To increase confidence in the 2 out of 3 DA predictions, borderline ranges were statistically derived (Gabbert et al., 2022) from the validation study data for each of the three *in vitro* methods (OECD 2021a). A 2 out of 3 prediction is considered to be of high confidence only if at least two test methods used in the DA give concordant predictions and the results fall outside the established borderline ranges. Results falling within the borderline ranges are of lesser confidence and should be used together with additional evidence to conclude on the presence or absence of sensitisation potential.

The level of confidence in the ITS DAs prediction is assigned based on the applicability domain of the individual information sources. In the case of the DPRA and h-CLAT methods, the limitations are described in the individual OECD TGs. For the in silico component of the DAs, the applicability of the individual in silico predictions is provided by the respective protocols. In the case of Derek Nexus (ITSv1), all positive predictions are within the applicability domain. Negative predictions are in domain unless they contain misclassified and/or unclassified features indicating either the presence of a molecular fragment present in known sensitisers but not alerted for by Derek Nexus or the presence of a fragment that has not been observed in publicly available data but that is present in proprietary data (Chilton et al., 2018). For the ITSv2, an OECD Toolbox automated workflow, specifically developed for the defined approaches (Yordanova et al., 2019) and implemented in the OECD Toolbox version 4.5, provides information on the applicability of the prediction based on three different layers, 1) parametric, 2) structural and 3) mechanistic. Depending on the toolbox prediction approach, i.e. read across or profiling prediction, and the prediction outcome, i.e. positive or negative, one or more of these layers are taken into account to define the overall applicability domain for a specific prediction.

## Predictive performance of the defined approaches

**FIGURE 3:**
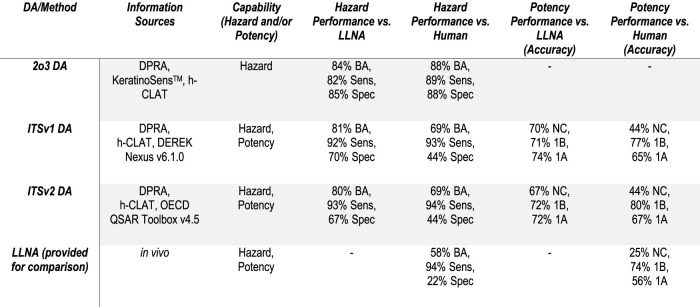
Summary Performance of the Defined Approaches. For hazard performance, sensitivity (Sens) is the true positive rate, specificity (Spec) is the true negative rate, and balanced accuracy (BA) is the average of sensitivity and specificity. For potency performance, accuracy reflects correct classification rate within each UN GHS sub-category.

The predictive performance of the three DAs included in the OECD Guideline was evaluated for discriminating between sensitisers and non-sensitisers, and for the ability of the ITS DAs to predict UN GHS potency sub-categories: 1A, 1B, or “not classified” (NC) (Figure 3). For these analyses only high confidence predictions were used to evaluate the overall performance of the DAs.

The performance of the three DAs against the agreed LLNA hazard classifications showed balanced accuracies (average of sensitivity and specificity; BA) in the range of 80–84%, with sensitivities of 82–93% and specificities of 67–85%. The performance of the ITSv1 and ITSv2 DAs for UN GHS classifications based on potency categorization (high confidence predictions only, sub-category 1A, 1B, or NC) when compared to the agreed LLNA potency classifications yielded overall accuracies of 71%, overall balanced accuracies of 78% (ITSv1) or 77% (ITSv2), and balanced accuracies within a predicted sub-category or NC ranging from 72–81% (ITSv1) or 71–80% (ITSv2). There were no strong sensitisers (1A) that were incorrectly predicted as being a non-sensitiser (NC) or vice versa.

When evaluated against the human reference data the performance of the DAs for predicting skin sensitisation hazard showed balanced accuracies (BA) in the range of 69–88%, with sensitivities of 89–94% and specificities of 44–88%. The performance of the ITSv1 and ITSv2 DAs for UN GHS skin sensitisation potency classification yielded overall balanced accuracies of 72% (ITSv1) or 73% (ITSv2), and balanced accuracies within a predicted sub-category or NC in the range of 68–79% (ITSv1) or 69–79% (ITSv2). When contrasted with the performance of the LLNA against the human reference data (58% BA for hazard and 64% BA for potency, overall), each of the DAs included in GL 497 showed equivalent or superior performance to the reference standard animal test.

An additional analysis was performed to characterise to what extent the DAs correctly identify chemicals that need to be activated either through abiotic activation (pre-haptens) and/or through biotic (enzyme-mediated) mechanisms (pro-haptens) to acquire skin sensitisation potential (OECD 2021b). In the dataset of 168 chemicals, there were 29 chemicals that are considered putative pre/pro haptens, and all of them were positive in the LLNA. Of these, only nine chemicals had human data available and all were found to be sensitisers in humans. Since all chemicals in the subset were positive *in vivo*, consequently no specificity or accuracy were calculated. ITSv1 and ITSv2 show excellent performance for pre/pro haptens as they predict essentially all chemicals correctly, with only one inconclusive result for ITSv2. The 2o3 shows a sensitivity of 83% against this set of chemicals, but with more inconclusive results than the other two DAs (*N* = 6 vs. *N* = 1). Overall, the DAs showed improved performance for predicting pre- and pro-haptens compared to the individual methods.

## Conclusion

The assessment of the skin sensitisation potential and potency categorisation of chemicals represents a standard requirement across many legislative sectors globally (Daniel et al., 2018). Progress has been made over the past decade in the validation and translation into international standards of in chemico and *in vitro* methods, each addressing a specific mechanism of the process of acquisition of sensitisation as represented by the AOP. Despite the uptake of such methods by specific regulations such as REACH in the EU, for the last several years information from these methods had to be used in the context of a weight-of-evidence approach since none of them is regarded sufficient to fully substitute for LLNA.

The application of defined approaches to combine these information sources overcomes the limitations of the individual methods, both in terms of predictivity and mechanistic coverage. Furthermore, DAs use a fixed data interpretation procedure to integrate the individual results, avoiding the use of expert judgment on an ad hoc basis in deriving the final prediction.

An unprecedented set of reference data were curated and applied for the assessment of the DAs in GL 497. The evaluation of the DAs has proven that they provide an equivalent level of protection than the LLNA for the set of chemicals evaluated, and can therefore replace the need for the animal tests for the purpose of hazard identification and potency sub-categorization of chemicals. Although testing of mixtures is currently not within the applicability domain of the DAs covered by Guideline 497, efforts are ongoing to adapt the protocols of the individual methods and expand the applicability of the DAs to the testing of multiconstituent substances. For example, the inclusion of a gravimetric approach in the DPRA for testing substances for which no molecular weight is available has been proposed and is under discussion at the OECD.

Guideline 497 on DAs for skin sensitization represents a first-of-its-kind product for the OECD, and sets a precedent for other human biology-based integrated testing strategies to come for a range of endpoints. As with all test guidelines, predictions obtained with DAs should nevertheless be used considering all existing available relevant information in the respective hazard and risk assessment frameworks.
